# Simulation study on the effects of cancellous bone structure in the skull on ultrasonic wave propagation

**DOI:** 10.1038/s41598-021-96502-5

**Published:** 2021-09-02

**Authors:** Itsuki Michimoto, Kazuki Miyashita, Hidehisa Suzuyama, Keita Yano, Yasuyo Kobayashi, Kozue Saito, Mami Matsukawa

**Affiliations:** 1 Doshisha University, Kyotanabe, Japan; 2 Nara Medical University, Kashihara, Japan

**Keywords:** Medical research, Engineering

## Abstract

The transcranial Doppler method (TCD) enables the measurement of cerebral blood flow velocity and detection of emboli by applying an ultrasound probe to the temporal bone window, or the orbital or greater occipital foramina. TCD is widely used for evaluation of cerebral vasospasm after subarachnoid hemorrhage, early detection of patients with arterial stenosis, and the assessment of brain death. However, measurements often become difficult in older women. Among various factors contributing to this problem, we focused on the effect of the diploe in the skull bone on the penetration of ultrasound into the brain. In particular, the effect of the cancellous bone structure in the diploe was investigated. Using a 2D digital bone model, wave propagation through the skull bone was investigated using the finite-difference time-domain (FDTD) method. We fabricated digital bone models with similar structure but different BV/TV (bone volume/total volume) values in the diploe. At a BV/TV of approximately 50–60% (similar to that of older women), the minimum ultrasound amplitude was observed as a result of scattering and multiple reflections in the cancellous diploe. These results suggest that structural changes such as osteoporosis may be one factor hampering TCD measurements.

## Introduction

The vertebrate skull has a complicated structure, consisting mainly of the following three layers: the outer cortical bone, the diploe (cancellous bone), and the inner cortical bone. The heterogeneous and anisotropic characteristics of these layers facilitate complex ultrasound propagation; however, recent advances in transcranial ultrasound transmission have provided new noninvasive therapeutic and diagnostic techniques for brain diseases^1,2^.

Some ultrasonic studies have assumed isotropic plate models, which do not accurately represent the actual structural and material characteristics of the skull^3,4^. For ultrasound irradiation to the skull with its complex characteristics, it is important to achieve precise control. Therefore, the heterogeneity and the structure of the skull has been investigated using computed tomographic (CT) data in relation to ultrasonic wave propagation^5,6^.

Among many diagnostic techniques for brain diseases, the transcranial Doppler (TCD) method is an approach that involves the application of an ultrasonic probe to the temporal bone window or the orbital or greater occipital foramina to evaluate cerebral blood flow velocity and detect emboli. This approach was first reported by Aaslid et al. in 1982^7^. Since then, the TCD method has been widely used in clinical practice for the evaluation of cerebral vasospasms after subarachnoid hemorrhage, early detection of thrombus in patients with arterial stenosis or left ventricular assistive heart^8^, and determination of brain death^9^. TCD usually uses focused and pulsed ultrasonic waves with a frequency of approximately 2 MHz^10,11^. TCD measurements can also be performed through the temporal acoustic window (TAW) to observe the arteries of the circle of Willis^12^. However, as Marinoni et al. reported, an inadequate TAW is a considerable problem for clinical use of TCD^13^, especially for older patients with cerebrovascular diseases^14^, although the use of an echo contrast agent improves the condition^15^. Halsey also observed a higher prevalence of inadequate TAWs in older subjects and in females^16^, while Hashimoto et al. reported that TCD measurements were often difficult in older Japanese women^17^. The rates of inadequate TAWs range from 9.8 to 29%^16–19^. Lin et al. also noted that the failure rate when using the temporal bone window for transcranial color-coded sonography was high in older women in Taiwan^20^. The European Federation of Neurological Societies (EFNS) task force guideline on neuroimaging in acute stroke states that TCD was hampered by the 10% to 15% rate of inadequate TAW most commonly seen in Blacks, Asians, and older female patients^21^. Similar results were found in Brazil^22^, and Doppler signals cannot be acquired in up to 38% of Amerindians because of an inadequate TAW^23^. The problem of acoustic windows is thus considered to be one of the major technical limits in TCD ultrasonography.

One possible reason for inadequate TAWs may be the morphological changes in the skull that accompany aging. Tsivgoulis et al. noted that an inadequate TAW was related to the thickness and porosity of the temporal bone, which attenuates ultrasound energy transmission^24^. Kwon et al. also reported that the thickness and inhomogeneity of the temporal bone, as well as factors related to age, sex, and hypercholesterolemia, were strongly correlated with TAW failure^25^. Kaito reported that the thickness of the skull increases in Japanese older individuals^26^. Interestingly, the total thickness of the skull increases with age, especially in older women; however, the density of the bone decreases^27^. This results in decreased thickness of the outer and inner layers, with a consequent increase in the thickness of the diploe (cancellous bone)^28^. It is well known that the bone volume fraction in the cancellous bone decreases with the progression of osteoporosis in older adults^29–32^. An excessive decrease in the bone volume fraction may result in strong ultrasound scattering and considerable attenuation of the observed ultrasonic waves. However, to the best of our knowledge, there has been no discussion in the literature about how cancellous bone structure changes caused by osteoporosis affect the TCD method.

In this study, we focused on the effects of the cancellous bone structure on ultrasonic wave propagation by analyzing the propagation phenomenon in skull models. The cancellous structure is anisotropic and complex. As can be seen in the CT images^5^, clear trabecular alignment in the thickness direction can be seen. In addition to this alignment, Murashima et al. reported a strong trabecular alignment in an anteroposterior direction in swine skulls^33^; this may also exist in the human skull and may influence ultrasonic wave propagation. Of course, bone material properties may also change as a result of age and lifestyle diseases in older adults^34,35^. Therefore, we have only focused in this study on the structural changes in the diploe caused by age. For this purpose, 2D digital partial skull models of the area near the temporal window were created. The bone volume / total volume (BV/TV) values of the models were different, but the structure was similar. These models are useful for checking the effects of BV/TV without structural changes. Finite-difference time-domain (FDTD) simulation was applied to these digital models to assess ultrasonic wave propagation in the thickness direction, which provides similar conditions to the TCD method.

## Model and method

### 2D digital temporal bone model

**Figure 1:**
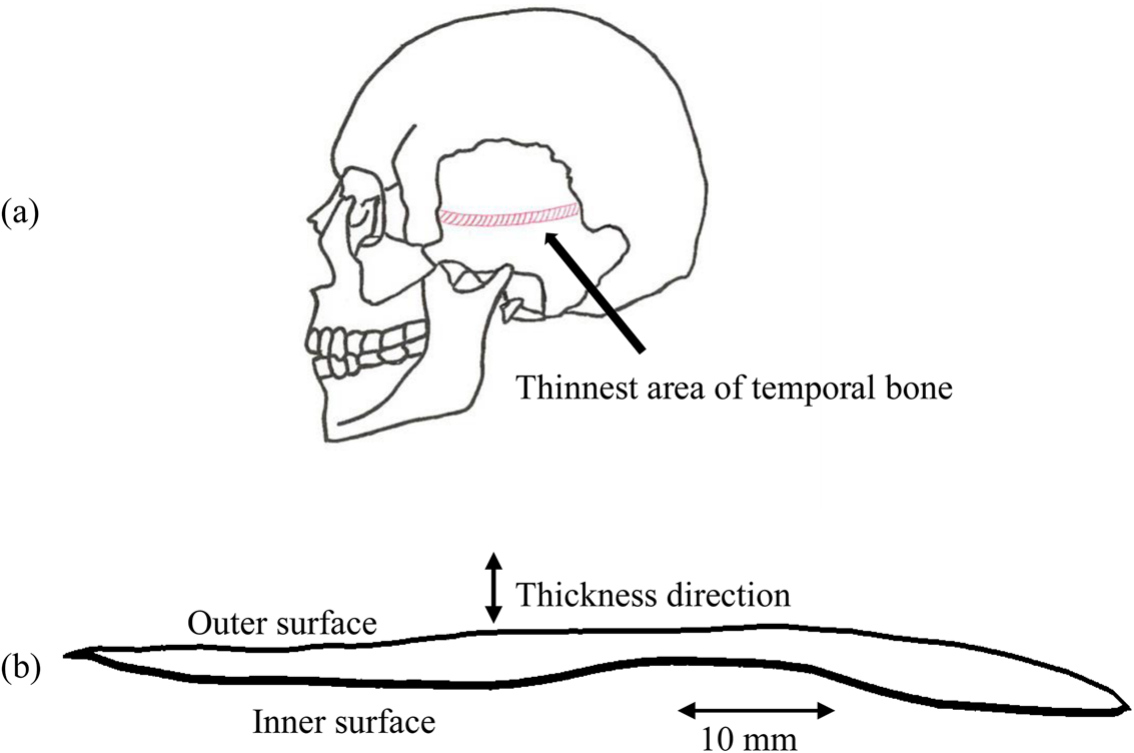
Position of the selected part of the skull and a cross-section view of the thinnest part of the left temporal bone.

A temporal skull bone section was created from a polygon model of the head of a healthy adult^36^. Figure 1a shows the position of the left temporal bone. Figure 1b shows a cross-sectional view of the thinnest region of the skull targeted by the TCD measurement. The internal structure of the cancellous bone consists of a network arrangement filled with bone marrow. An important parameter of the cancellous bone is BV/TV, which represents the ratio of the actual bone volume to the total volume of the sample.

**Figure 2:**
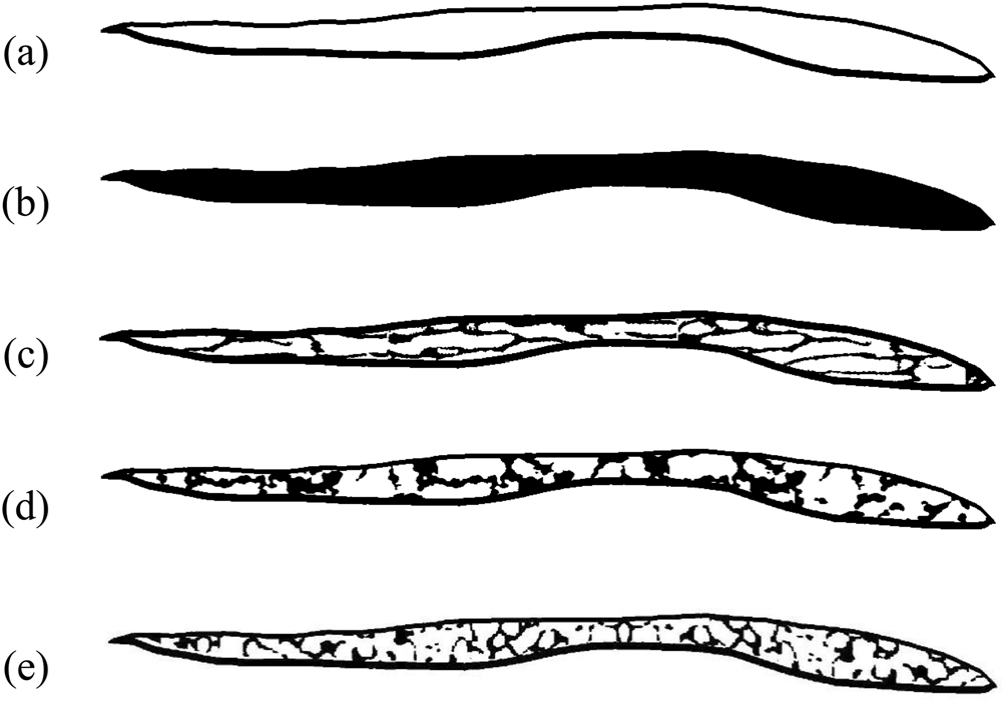
Five bone models. (**a**) Empty model, (**b**) uniform model, (**c**) parallel equine model (sample A), (**d**) swine model (sample B) and (**e**) human model (sample C). Approximate length of the models is 65 mm.

**Figure 3:**
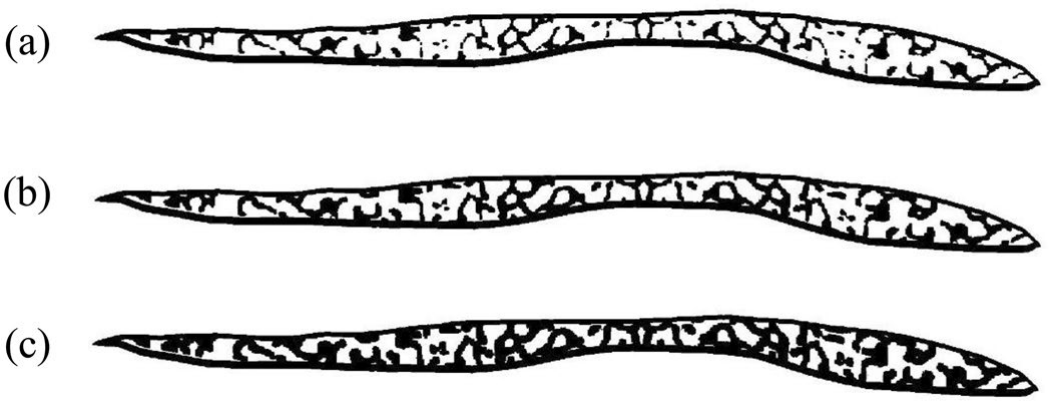
Three bone models of sample C with different BV/TV. (**a**) 50%, (**b**) 60% and (**c**) 70%.

In this study, five types of models were fabricated as shown in Fig. 2. In the empty model, the diploe was filled with water to mimic bone marrow. In the uniform model, the diploe was filled with bone (BV/TV = 100%). In sample A, the trabecular bone was aligned nearly parallel to the cortical bone layer, and the diploe was filled with water. The trabecular bone was created from a CT image of the actual cancellous bone in an equine femur, where the trabeculae almost align along the outer and inner surfaces of the bone. The trabeculae showed a high degree of anisotropy (DA; 2.5)^37^. In sample B, the trabeculae were mostly aligned perpendicular to the cortical layer. This was created from a CT image of the cancellous bone of a swine skull. Samples A and B were fabricated to check the anisotropic character of the cancellous part of the bone. Sample C was fabricated from a reference CT image of a human skull^5^. Figure 2 shows examples of bone samples with BV/TV values of 50–60%, which is similar to the BV/TV of an older adult human skull^38^. Using a bone model derived from X-ray CT images, Nagatani et al. fabricated various BV/TV models by changing the thickness of the trabeculae with an image processing technique^39^. Making use of the CT images shown by grayscale (0–255), various BV/TV models were also created in this study by choosing several different grayscale thresholds. However, control of the grayscale did not have a strong influence on the alignment direction of the trabeculae. Figure 3 shows model samples in the case of sample C. Total BV/TV and the partial (cancellous section) BV/TV values of all models are shown in Table 1.

**Table 1:** BV/TV values of bone models.

Sample A		Sample B		Sample C	
BV/TV[%]	BV/TV[%] Cancellous part	BV/TV[%]	BV/TV[%] Cancellous part	BV/TV[%]	BV/TV[%] Cancellous part
83	75	68	53	80	70
81	73	66	50	75	63
75	63	63	47	70	56
69	55	60	42	65	49
68	53	58	39	60	41
67	51	56	35	55	34
64	48	53	31	50	27
62	44	50	27	45	19
60	42	48	25	40	12
59	41	46	22	35	5
58	38	44	18	-	-
56	36	-	-	-	-
54	32	-	-	-	-
52	30	-	-	-	-

### FDTD simulation and conditions

A simple elastic 2D FDTD simulation was performed to simulate longitudinal and shear wave propagation in the model^40,41^. Similar to previous studies by our group^42,43^, an in-house FDTD source code was used. In this code, wave velocities in the bone matrices were fixed and absorption was not considered. One reason for this assumption was to focus on the effects of BV/TV and structure on the wave propagation, which may be strong in the cancellous bone in the diploe. In fact, Nagatani’s comparative study showed that the simulated results were similar to the experimental waves in the cancellous bone, although the absorption in the bone matrices was not considered^44^. Another reason for this assumption is the age and frequency dependence of the wave properties in bone. It is known that the wave velocities in new and mature bones are different at the bone matrix level^34^. Yasui et al. reported that the velocity decreases in diabetic bone, implying that the properties of bone change as a result of lifestyle-related disease^35^. The in vivo study of Talmant et al. showed a clear decrease in the first arriving signal (FAS) velocity in the cortical bone of older adults^45^. These velocity results may also imply changes of absorption in the bone matrix. These changes in wave properties should also be considered in the simulation; however, because precise measurement of wave properties in the bone matrices is difficult, we fixed the velocity values and focused on structural effects in this study.

These models were homogeneous with the bone mass density of 2000 kg/m^3^^46,47^. There are several discussions on the wave properties in the skull^48,49^. We used the velocities of longitudinal wave (3000 m/s) and shear wave (1500 m/s), respectively, following the studies of Pinton et al.^5^. The estimated Poisson’s ratio was 0.33, which was a reasonable value for bone^47^. In the FDTD simulation, wave equations were computed numerically using the central difference method. The stress and particle velocity were both calculated alternately in the spatial and time domains, which is called “the leapfrog method.” Higdon’s second-order absorption boundary condition was implemented at the edges of the simulation area as an absorption layer^50^.

**Figure 4:**
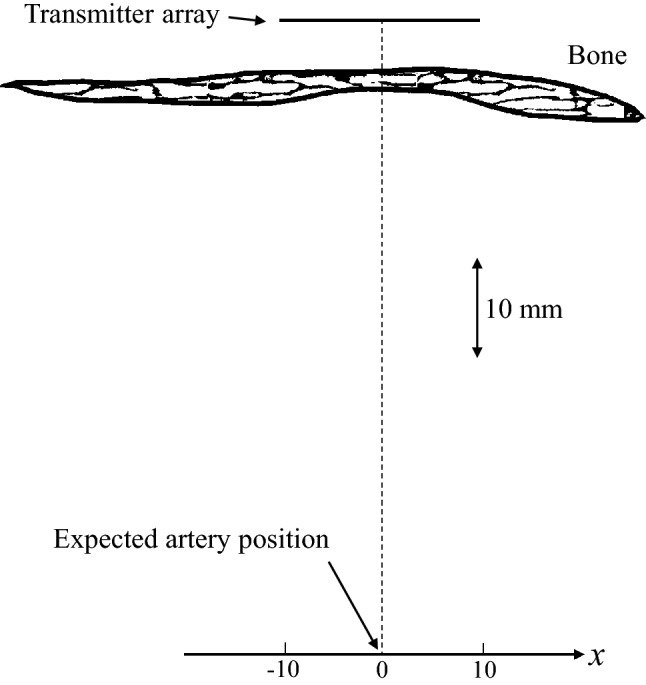
Simulation conditions. The center of the transmitter array and observed positions were at *x* = 0. They are arranged parallel with distance of 60 mm. All parts are immersed in water to mimic the soft tissues.

Figure 4 shows the simulation conditions used. A transmitter array (length = 20 mm) was placed outside the bone model and in front of the thinnest part of the bone. The transmitter was an array of 100 transducers that controlled the phase to focus the wave near the artery position (*x* = 0 mm). The radiated waveform from the transmitter was a single sinusoidal wave at 2 MHz with a Hann window. The bone model was placed 5 mm from the transmitter considering the thickness of the skin and the temporalis muscle. In this study, we considered that the temporalis muscle had isotropic elasticity, because ultrasound usually propagates perpendicular to the uniaxial alignment direction of the muscle. The propagating wave was observed at receivers on the *x* axis, which was 60 mm from the center of the transmitter array. This is the expected position of the middle cerebral artery or the posterior cerebral artery, which are usually measured from the TAW by TCD^51^.

The spatial and time resolutions in the simulations were 14 μm and 2.5 ns, respectively. These resolutions satisfied the Courant stability condition^52^. In this simulation, the bone model and transmitters were all immersed in water to mimic the surrounding soft tissues and bone marrow. The longitudinal wave velocity and density of water were 1500 m/s and 1000 kg/m^3^, respectively.

## Results and discussion

**Figure 5:**
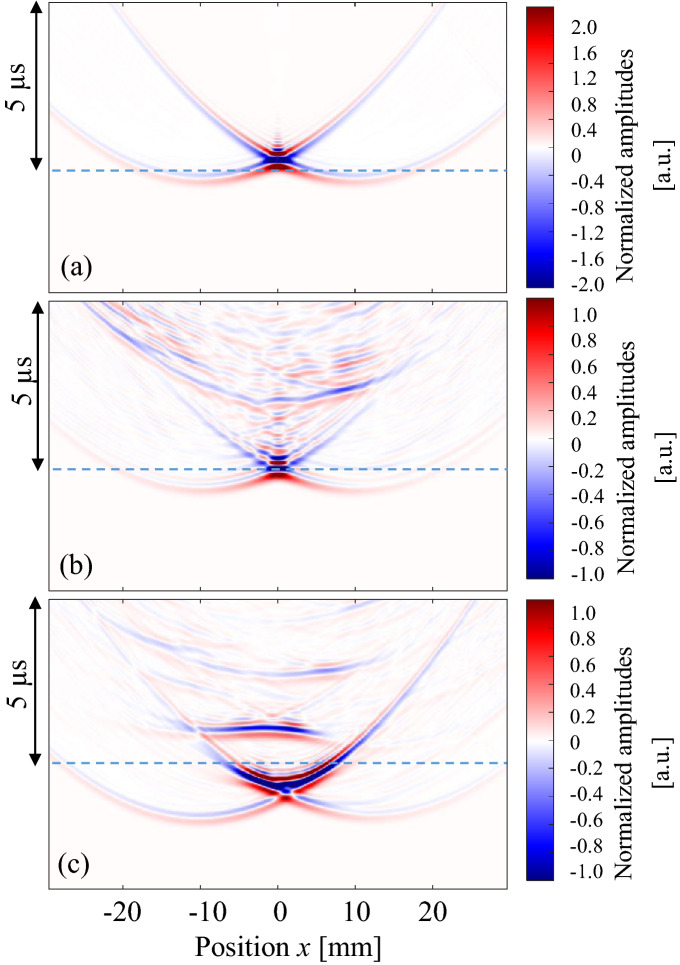
Observed waves at different positions. Ultrasonic waves propagated through (**a**) water only, (**b**) the empty model and (**c**) the uniform model. Dashed line indicates the arrival time of the wave which passed through water only.

Figure 5 shows the observed waves that propagated in (a) water only, (b) the empty model without the cancellous section, and (c) the uniform model (BV/TV = 100%). Here, all amplitude values were normalized by the maximum amplitude of the wave that passed through the uniform model. In Fig. 5a, the highest amplitude was observed on the acoustic axis (*x* = 0 mm) and the axis-symmetric wave propagation was clearly found. The figure shows that the array transmitter successfully focused the ultrasound at the artery position (*x* = 0 mm). The arrival time around *x* = ± 10 mm was the shortest where very weak waves were observed. This is because the wave that was radiated from each end of the transmitter array reached these positions first. In Fig. 5b,c, the wave fronts arrived earlier because of the higher wave velocities in bone. In the empty model in Fig. 5b, small waves were found approximately 2.3 μs later that the initial wave front. This is the internal reflected wave at the cortical shell. In the uniform model seen in Fig. 5c, the second wave was observed after the focusing waves, which was the reflected wave in the bone sample. The delay time of the second wave (approximately 2 μs) is reasonable considering the bone thickness (approximately 3 mm) and the wave velocity (3000 m/s). Additionally, in the empty model, the wave was focused mostly near the artery; however, the maximum amplitude was not observed on the acoustic axis (*x* = 0) in the uniform model, showing only a very small shift. The symmetrical wave propagation changed as a result of the bone shape in the propagation path. This indicates that ultrasound focusing was clearly affected by the complex bone shape, which has a partial concave structure.

**Figure 6:**
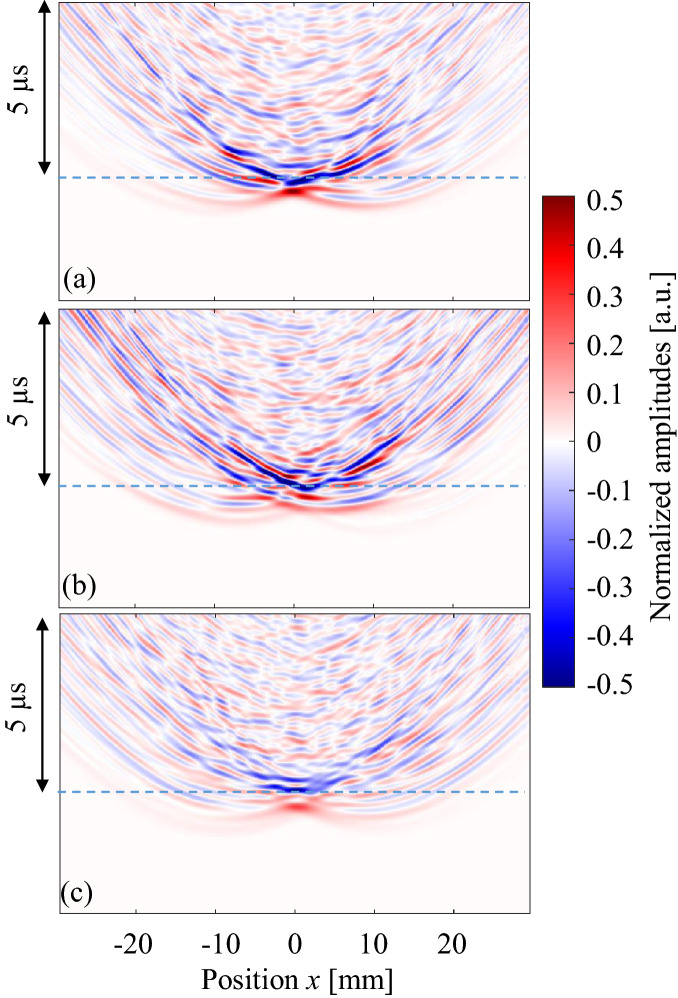
Observed waves at different positions. Ultrasonic waves propagated through (**a**) sample A, (**b**) sample B and (**c**) sample C. BV/TV values of these samples were 55–56%. Dashed lines indicate the arrival time of the wave which passed through water only.

Next, observed waves that passed through samples A, B and C are shown in Fig. 6. The BV/TV values of these samples were 55–56%. Here, all amplitude values were normalized by the maximum amplitude of the wave that passed through the uniform model. It can be seen that the amplitudes of the waves became smaller as a result of multiple scattering of the waves in the cancellous structure. Complicated wave propagation can be seen in all figures, where the axis-symmetrical characters are being lost, especially in Fig. 6b. One interesting result in Fig. 6 is the arrival time of each wave. Although the BV/TV values were similar, the arrival times on the acoustic axis (*x* = 0 mm) were different because of the structure. In sample A, the wave arrived 0.5 μs faster than the wave propagated in water only. In sample C, the arrival time difference was approximately 0.7 μs. Sample B recorded the fastest arrival time (0.95 μs). These arrival times may have resulted from structural differences. In sample A, most trabeculae were aligned nearly parallel to the cortical bone layer, whereas in sample B, the thick trabeculae were aligned in the thickness direction in the thinnest area. Sample C had a mixed kind of structure. It is also interesting that large amplitudes were found at the arrival time of the water wave in sample B, signifying that most of the wave passed though the water among the trabeculae. It is known that the ultrasonic longitudinal waves in the MHz range often separate into fast and slow waves in cancellous bone; this is known as the two-wave phenomenon^32^. This phenomenon was experimentally confirmed for the first time in bovine cancellous bone^47^, and then in human cancellous bone in femora^53^. Although the wave propagation direction was different, Murashima et al. also reported the two-wave phenomenon in the diploe of a swine skull^33^. The partial BV/TV of the cancellous part in sample B was 35% in Fig. 6b. Mizuno et al. showed a clear two-wave phenomenon in human cancellous bones with BV/TV values ranging from 15 to 35%^53^. In the two-wave phenomenon, the slow wave mostly propagates in the liquid part of the cancellous bone and the propagation speed is almost the same as that in liquid. Therefore, the comparatively large amplitude of the waves in sample B may indicate the existence of a slow wave.

**Figure 7:**
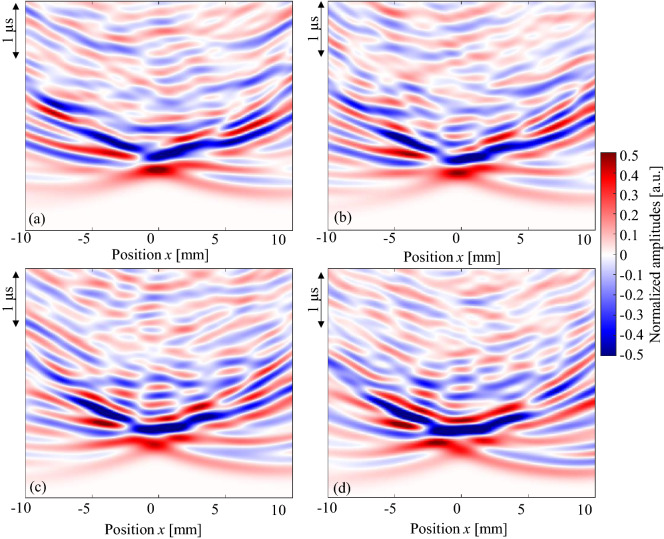
Observed waves at different positions. Ultrasonic waves propagated through models of sample A with different BV/TV values; (**a**) 56%, (**b**) 62%, (**c**) 68% and (**d**) 73%.

**Figure 8:**
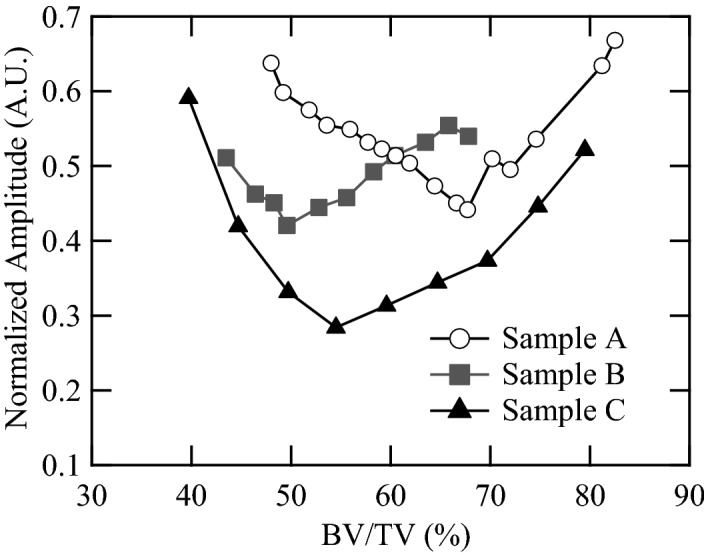
Maximum amplitudes of the observed pulse waves as a function of BV/TV. The amplitudes were normalized by the maximum value of the observed waves which passed through the uniform model.

**Figure 9:**
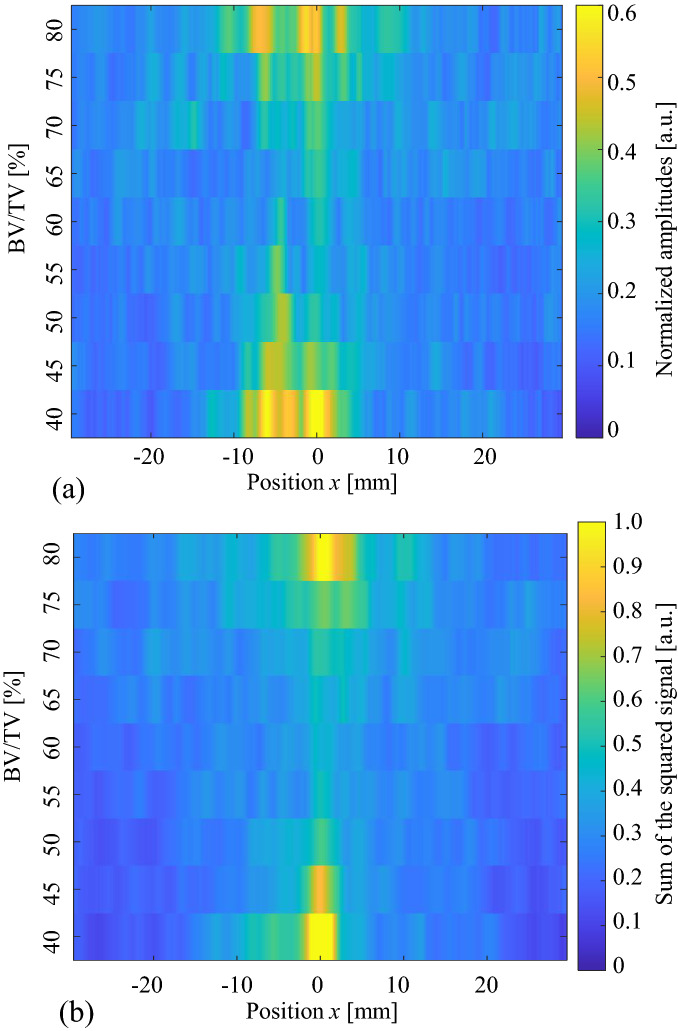
(**a**) Maximum amplitudes of the observed pulse waves and (**b**) sum of the squared signal as a function of BV/TV and observed positions (sample C). The amplitudes were normalized by the maximum value of the observed waves which passed through the uniform model. The squared signal data were also normalized by the maximum value.

Figure 7 shows enlarged images near the water front (sample A). Clear non-axis-symmetric wave propagation and changes in the maximum amplitude positions can be seen. The maximum amplitudes changed because of the BV/TV values. Figure 8 summarizes the maximum amplitudes as a function of BV/TV. All samples (samples A, B, and C) showed minimum values in the range of BV/TV from 50 to 70%, and approximately 27–56% in the cancellous part (Table 1). In sample C, in which the pore size in the cancellous part seems comparatively small, the amplitude changes were dynamic, demonstrating the stronger effects of cancellous bone. These characteristics are also clear in Fig. 9, which shows the maximum amplitudes and the sum of the squared signals at all measured positions. Here, the sum of the squared signal means the integrated values of the squared signal, which indicates changes in the total intensity of the ultrasound. Figures 5 and 6 show several late small waves that were observed after the initial waves. The small waves may have originated from scattering and multiple reflections. In the human bone model (sample C), the sum of the squared signal also became small in the range of BV/TV values from 50 to 70%. This implies that the energy passing through the bone decreases as a result of scattering and multiple reflections. In sample C, the small shift in the maximum amplitude positions can also be seen; however, the sum of the squared signal was strongest in the center. These values were small in the model with a BV/TV value of 55–70%, indicating that the amplitude and intensity minima exist in this range. For TCD measurements, we usually use pulse ultrasonic waves, where the maximum amplitude seems more important than the total intensity of the wave, which is in contrast to ultrasonic treatment modalities such as high intensity focused ultrasound (HIFU)^54^.

Larsson et al. reported that the BV/TV in cancellous bones decreased with age^38^. They showed that the BV/TV in the skull bone of a 70-year-old woman was approximately 62%, whereas that of a 50-year-old woman was approximately 82%. Our results indicate that the decrease in BV/TV with age may prevent ultrasounds from reaching the arteries. This may make it difficult to use TCD measurements for older women. In the skull, the main alignment of the trabeculae is considered to be in the direction of the thickness^29^. Despite structural differences, all samples showed a clear decrease in the maximum amplitude at a BV/TV of approximately 50–55%; these values are reasonable to the values reported in older patients.

As the BV/TV values increased, the maximum amplitude position near the wave front gradually shifted from the acoustic axis (*x* = 0 mm), implying that it may be difficult to control wave focusing because of the characteristics of the cancellous bone. This could be attributed to the slight tilt of the trabeculae in the thickness direction. Consequently, ultrasound may be refracted in the bone model. Yamashita et al. reported that the bone trabecular alignment affected the direction of ultrasound propagation^55^. Thus, the ultrasound did not focus strongly near the artery. Similar scattering phenomena and refraction of the waves may occur in an actual skull bone.

**Figure 10:**
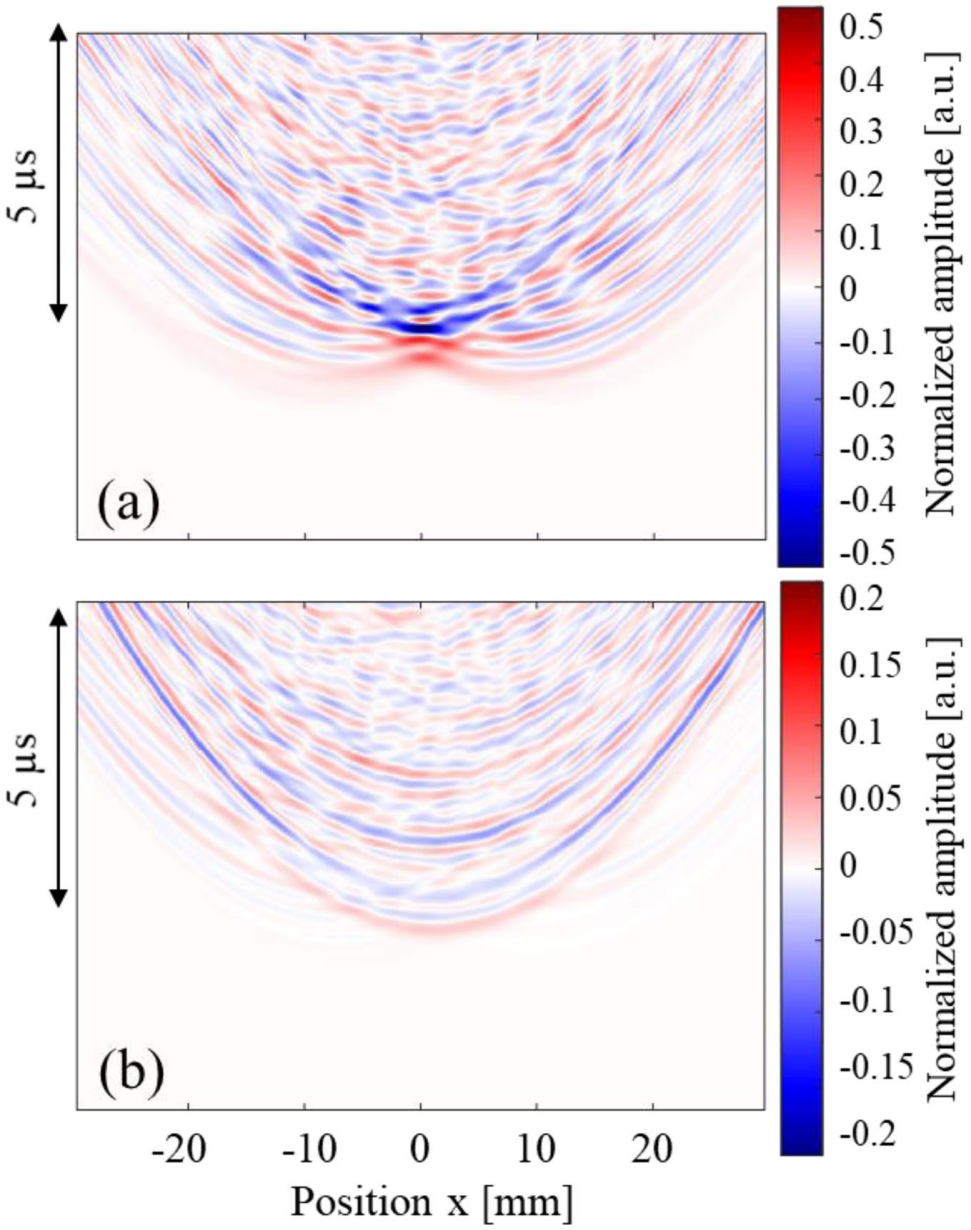
Maximum amplitudes of the observed pulse waves (sample C, BV/TV = 50%). (**a**) Cancellous part was filled with water. (**b**) Cancellous part with no water.

In addition to the effects of osteoporosis, Del Brutto et al. noted the existence of aberrant pneumatization, which would produce dramatic changes in the ultrasound transmission^23^. Figure 10 shows the comparative results of wave propagation in sample C with (a) and without (b) water in the diploe (cancellous part). For the simplicity of the simulation, the cancellous part was filled with vacuum because the acoustic impedance of air is negligible. The amplitudes dramatically decreased in the bone model without water. This may be an important problem with TCD measurements. Without water in the cancellous section, the wave which propagated only along the trabeculae, was very weak, and did not focus, depending on the structure.

This study demonstrates the possible effects of ultrasound scattering in the cancellous section of the diploe layer in the skull. Because older women tend to have a thicker diploe layer, the effects seem strong. To compensate for these effects for TCD measurements and ultrasonic brain therapy, it is important to achieve appropriate ultrasound focusing with adjustment of the phase of radiated ultrasound considering the complex structure. One possible solution is time reversal processing^56^.

Because this study focused on the structural effects on ultrasonic wave propagation in the TAW, we did not consider absorption of ultrasound in the simulation. The material absorption will decrease the wave amplitude and intensity. Age-dependent ultrasonic wave properties (velocity and absorption) should be carefully considered in future studies. To understand the complicated wave propagation in the skull, precise 3D simulation is also necessary by comparing with real experimental studies of skull bones.

## Conclusion

In this study, the effect on ultrasound propagation of structural changes in the skull bone was investigated by FDTD simulation. By changing the BV/TV in 2D human temporal bone models, we found a minimum value for the ultrasound amplitudes caused by scattering and reflection. Although three different bone models were used, ultrasound was strongly attenuated in the bone around samples with BV/TV values of 50–70%, which was similar to the BV/TV value of the diploe in the skull of older women. Structural changes in the skull may affect ultrasonic penetration into the brain and may be one factor that can explain the difficulty in measuring TCD in older adults. This study indicates that the effect of skull structure on ultrasound propagation is an important factor for future brain therapy and diagnosis.
